# Epidemiologic investigation of a family cluster of imported ZIKV cases in Guangdong, China: probable human-to-human transmission

**DOI:** 10.1038/emi.2016.100

**Published:** 2016-09-07

**Authors:** Yingxian Yin, Yi Xu, Ling Su, Xun Zhu, Minxia Chen, Weijin Zhu, Huimin Xia, Xi Huang, Sitang Gong

**Affiliations:** 1 Guangzhou Institute of Pediatrics, Guangzhou Women and Children's Medical Center, Guangzhou Medical University, Guangzhou 510623, China; 2 Department of Infectious Diseases, Guangzhou Women and Children's Medical Center, Guangzhou Medical University, Guangzhou 510623, China; 3 Key Laboratory of Tropical Disease Control (Sun Yat-sen University), Ministry of Education, Guangzhou 510080, China; 4 Department of Pediatric Surgery, Guangzhou Women and Children's Medical Center, Guangzhou Medical University, Guangzhou 510623, China; 5 Department of Gastroenterology, Guangzhou Women and Children's Medical Center, Guangzhou Medical University, Guangzhou 510623, China

**Keywords:** arbovirus, flavivirus, molecular epidemiology, Guangdong, Zika virus

## Abstract

Zika virus (ZIKV) is an emerging mosquito-borne flavivirus that can potentially threaten South China. A Chinese family of four returning from Venezuela to China was found to be positive for ZIKV when the youngest son's fever was first detected at an airport immigration inspection. They were isolated temporarily in a local hospital in Enping city, Guangdong province, where their clinical data were recorded and urine and saliva were collected to isolate ZIKV and to obtain viral sequences. All of them except the mother presented mild symptoms of rash and fever. Envelope gene sequences from the father, daughter and son were completely identical. Phylogenetic analysis demonstrated that this strain is similar to several imported strains reported in recent months, which are all clustered into a group isolated from 2015 ZIKA outbreaks in Brazil. Together with the climatic features in Venezuela, New York and Guangdong in February, it can be concluded that our subjects are imported cases from Venezuela. With the same viral sequence being shared between family members, neither direct human-to-human nor vector transmission can be ruled out in this study, but the former seems more likely. Although our subjects had mild illness, epidemiologists and public health officials should be aware of the risk of further expansion of ZIKV transmission by local competent vectors.

## INTRODUCTION

Although Zika virus (ZIKV) was first identified early in 1947 in Uganda, Africa, outbreaks in French Polynesia in 2013 significantly accelerated the spread of this virus to other parts of the world. ZIKV is a reemerging mosquito-borne flavivirus circulating in a wide range of regions including Africa, South America, and Asia.^1^ ZIKV infection can cause serious damage to the central nervous system, such as infant microcephaly and Guillain–Barré Syndrome.^2, 3, 4^ The virus has proven to be neurotropic in animals, and a recent experiment *in vitro* also showed that it can infect human neural progenitor cells derived from induced pluripotent stem cells.^5^ Another study showed that human dermal fibroblasts, epidermal keratinocytes and immature dendritic cells are also permissive to the most recent ZIKV isolates.^6^ An animal model of ZIKV infection has been established in AG129 mice by foot pad injection.^7^

By far, *Aedes aegypti* is considered the principal transmission vector of ZIKV,^8^ although *Aedes albopictus*, which caused several outbreaks of dengue fever in Guangdong Province of South China in the last two decades, may play a role in the spread of this virus because *A. albopictus* may be a competent vector.^9^ There are over 180 000 Chinese in Venezuela, which is one of the regions most heavily affected by ZIKV infection in South America.^10^ With frequent people shuttling between South America and Guangdong, there is a potential risk of spreading ZIKV to South China, where *A. albopictus* are active in densely populated communities.

In this study, a family of four flying from Venezuela to Guangzhou of Guangdong Province was found to be ZIKV positive in their peripheral blood. To gain a better understanding of transmission among communities, the phylogenetic relationship between the isolates from this family and others from diverse regions of the world was analyzed. Because this virus may be transmitted directly by body fluids,^11, 12, 13^ it was also necessary to explore this possibility in this family.

## MATERIALS AND METHODS

### Infected individuals, samples collection and ethic statements

**Figure 1:**
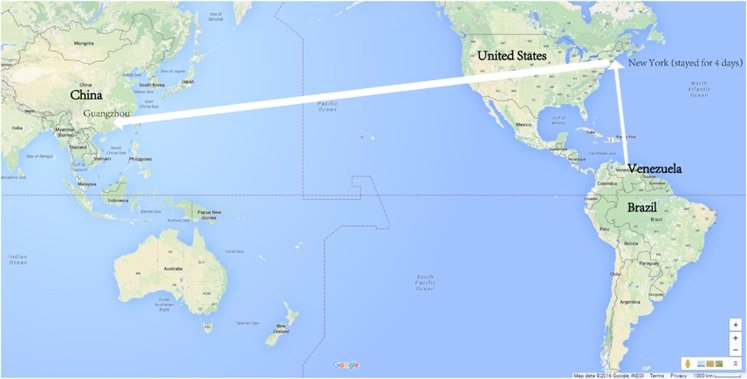
Route of the family traveling from an affected area to South China in late February 2016. On their way from Venezuela to Guangzhou, they had a layover in New York, where they stayed for four days.

Four hospitalized individuals from a family (father, mother, daughter and son) were diagnosed with ZIKV infection at Enping People's Hospital. This family had lived in Venezuela for more than two months before 20 February 2016; then they flew to New York on that day and stayed there for ~4 days. Finally, they flew from New York to Guangzhou, China on 24 February 2016 (Figure 1). These four infected individuals were first confirmed by real-time reverse-transcription polymerase chain reaction (RT-PCR) in Baiyun International Airport of Guangzhou, where the youngest one (the son) had developed fever, and the family was then isolated by the local department of public health. The infected individuals then lived in a shared ward (without other patients) in the infectious disease department of Enping People's hospital. The room had been screened against mosquitoes, and each bed was also covered with a bed net to prevent spreading by local competent vectors. At the time of hospitalization, the subjects' clinical history and results of a general physical examination, blood tests and routine urine tests were documented. The youngest one (a 6-year-old boy) was the first case whose manifestation was fever and maculopapules. Saliva, urine and peripheral blood were collected from the patients during the onset and recovery period and were stored at −80 °C. All samples were tested for ZIKV RNA by real-time PCR, and some urine was utilized to isolate virus. Informed consent was obtained from all patients before sample collection. The study protocols were reviewed and approved by the Scientific and Ethical Committee of Guangzhou Women and Children's Medical Center.

### RNA extraction

Before RNA extraction, urine samples were concentrated with Amicon Ultra-0.5 Centrifugal Filter Units with Ultracel-10 membrane (Millipore, Shanghai, China). The QIAamp Viral RNA Mini Kit (Qiagen, Hilden, Germany) was used to extract viral RNA from urine and saliva according to the manufacturer's instructions. RNA was eluted in 50 μl of AVE buffer and stored at −80 °C until use.

### RT-PCR and sequence analysis

Samples positive for ZIKV were selected to amplify genes encoding Envelope protein (E) and nonstructural protein 1 (NS1). Complementary DNA (cDNA) was synthesized from viral RNA by using the RevertAid First Strand cDNA Synthesis Kit (Thermo Fisher, Salt Lake City, USA) according to the manufacturer's instructions. Four pairs of primers were designed to generate overlapping DNA fragments covering the E and NS1 gene regions by using the software Oligo7.0 (http://www.oligo.net/.Supplementary Table S1). Polymerase chain reaction (PCR) was performed with Ex Taq HS DNA Polymerase (TaKaRa, Dalian, China) under conditions of initial heating of 95 °C for 3 min, followed by 30 cycles of 94 °C for 30 s, 56 °C for 30 s and 72 °C for 1.5 min. The sequences of the PCR products were identified by using the Sanger sequencing method.

### Phylogenetic analysis

Before phylogenetic analysis, the E and NS1 coding sequences of four individuals in our study were aligned with other reference sequences using Clustal 2.1 software (http://www.clustal.org/clustal2/). The reference sequences selected were those with highly similar BLAST (Megablast, http://blast.ncbi.nlm.nih.gov/Blast.cgi) scores. Phylogenetic trees were drawn using the maximum likelihood method in the Tamura-Nei model with gamma-distributed evolutionary rates in MEGA 7.0 (www.megasoftware.net).^14^ An initial tree was made automatically with the nearest-neighbor-interchange method. The gaps/missing data treatment was set as complete deletions. Bootstrap analyses with 1000 replications were utilized to determine confidence values for groupings within the phylogenetic trees. Other parameters were set to default style.

## RESULTS

### Clinical characteristics of four individuals hospitalized with ZIKV infection

**Figure 2:**
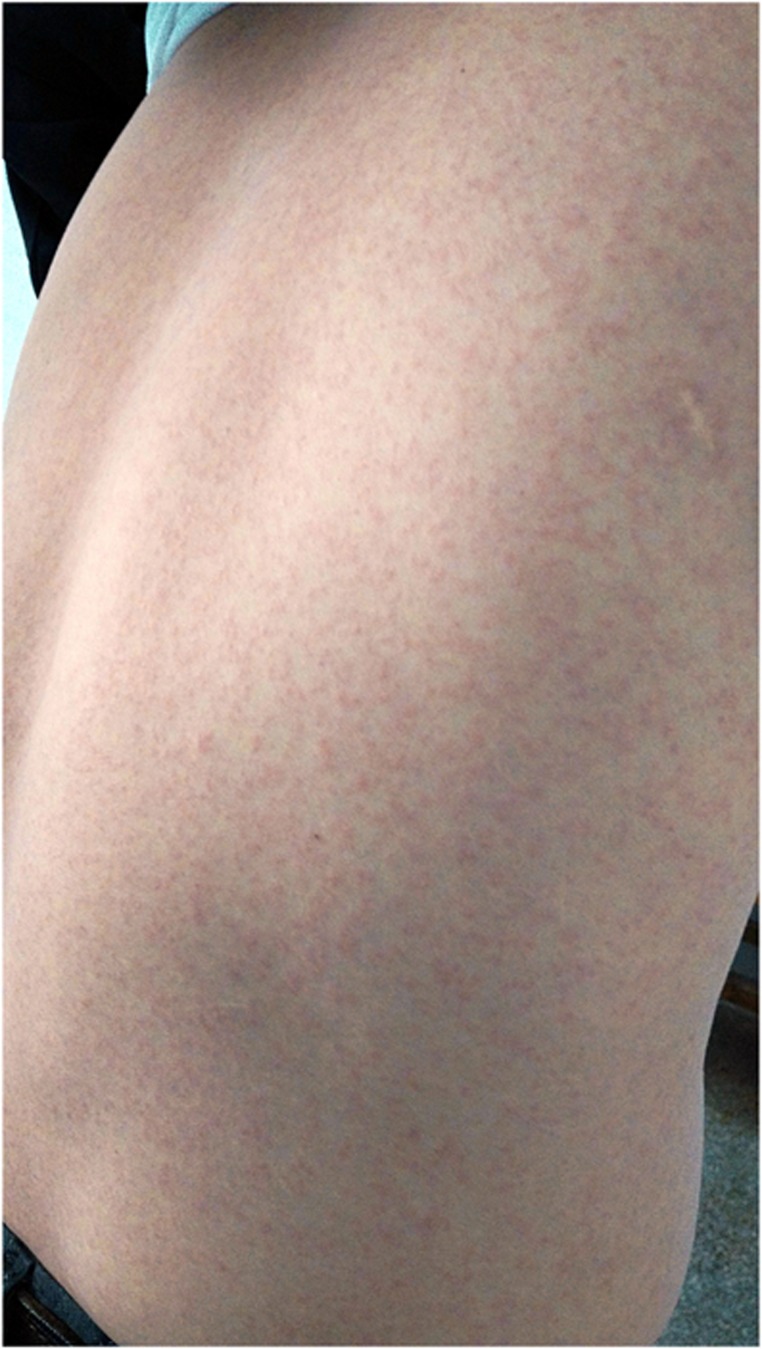
Rashes on the back of patient 1, who was infected with Zika virus.

**Figure 3:**
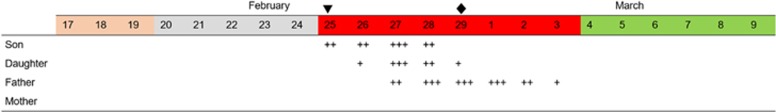
Inferred time axis ranging from probable biting by mosquitoes to release of ZIKV-infected family members. +, ++, +++ represent varying degree of rashes on the neck and back of patients. ▾ Time point when peripheral blood was positive for ZIKV using real-time PCR. ♦ Time point when urine or saliva was positive for ZIKV using real-time PCR. March 9 was the date of release from isolation.

The clinical characteristics of four individuals with ZIKV infection are summarized and compared in Table 1. In this family, whose members were living together for a long period before and after the first onset, the 6-year-old son was the first patient, and he had both fever and rash (maculopapules). Before returning to China, they had no symptoms of any infectious disease, but the son and the daughter both had a history of mosquito bites in Venezuela according to the father's memory, although the specific date of biting was not clear. The 8-year-old daughter was the second one who had symptoms, which included fever and rash, with the rash occurring on the second day after her brother had fever and rash. The 40-year-old father was the latest patient; the only symptom he had was a rash, and the rash was distributed uniformly on his neck and back (Figure 2). His neck rash first occurred on the third day after his son showed fever and rash. The 37-year-old mother did not have any symptoms during the whole period of observation. A time axis based on rash indicates the sequence, the time interval and the date of positive detection for ZIKV (Figure 3). The four family members had been isolated since 25 February 2016, and they were released on 9 March 2016. The clinical laboratory showed results that the overall symptoms of the four subjects were mild. The daughter and the son were subjected to laboratory blood tests twice and thrice, respectively (Table 1). Unlike most other viral infectious diseases, low white blood cell counts were not a common feature of our subjects.

### Detection of ZIKV in saliva and urine from four hospitalized patients.

Real-time PCR based on TaqMAN probes was used to detect ZIKV RNA. Among the four tested urine samples collected on 29 February 2016, patient 1 (father), patient 3 (daughter) and patient 4 (son) were found to be positive for ZIKV. Only patient 4 was positive for ZIKV in a saliva sample (detected on the night of 29 February 2016, when the samples were collected). The urine and saliva samples from patient 2 (mother) were all negative for ZIKV detection.

### Nucleic acid sequencing, alignment and phylogenetic analysis

**Figure 4:**
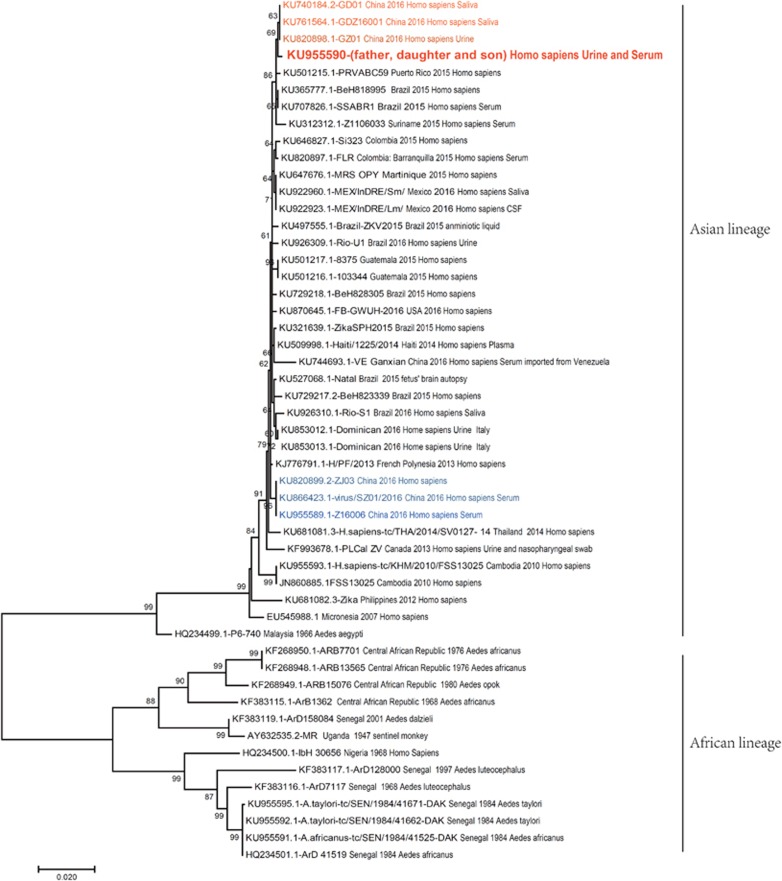
Phylogenetic tree based on E gene sequences of Zika virus isolates. E gene sequences from the father, daughter and son (Genbank: KU955590) in our study were used for alignment with other reference sequences. All isolates are indicated with related information (Genbank number, country, year and host, some with sample type). Phylogenetic trees were drawn using the maximum likelihood method by the Tamura-Nei model with gamma-distributed evolutionary rates in MEGA 7.0. An initial tree was made automatically with the nearest-neighbor-interchange (NNI) method. Gaps/missing data treatment was set as complete deletion. Bootstrap analyses with 1000 replications were utilized to determine confidence values for groupings within the phylogenetic trees. Other parameters were set to default style.

PCR products were successfully obtained only from patient 1, patient 3 and patient 4. After sequencing and fragment assembly, the E gene sequences of the three patients were found to be exactly the same. The E gene sequence of patient 1 is identical to Z16019 (GenBank: KU955590.1, submitted by Wu *et al*, Guangdong Provincial Center for Disease Control and Prevention), the viral sequence from the same patient. We found that all our sequences were similar to those of strains circulating in South America, especially Brazil (Figure 4). To further investigate the origin of the ZIKV isolated from our subjects, phylogenetic trees were constructed using the maxim likelihood method. The whole tree can be divided into African and Asian lineages, and all strains isolated from China belong to the Asian lineage. The strain HQ234499.1-P6-740, isolated from *A. aegypti* in Malaysia in 1966, is the earliest ancestor of the Asian lineage. The strains KU820899.2-ZJ03, KU866423.1-virus/SZ01/2016 and KU955589.1-Z16006 are isolated among imported cases from Samoa or Fiji, pacific islands near Asia. From the aspect of molecular evolution, they are at the ancestor level of French Polynesian strain KJ776791.1-H/PF/2013, which caused an outbreak of ZIKV infection in 2013. KU744693.1-VE Ganxian (imported from Venezuela) is another cluster located a long distance from the cluster of current imported Venezuelan strains in our study. Our strains from the family we are studying, as well as other strains isolated from imported cases in China during the same period of time, form a cluster near the Brazilian strains isolated in 2015. As there is a high bootstrap value at the node of this branch, together with enough evolutionary distance, HQ234499.1-P6-740 (isolated from *A. aegypti*) is an ideal outgroup strain in the Asian lineage.

## DISCUSSION

ZIKV infection is becoming a global public health concern since microcephaly cases were linked to ZIKV infection during pregnancy. So far, the data suggesting that microcephaly cases in Brazil might be linked with ZIKV infection are only epidemiological.^15, 16^ Growing evidence has indicated that ZIKV can damage the fetus, causing intrauterine growth restriction.^17, 18^ A recent report that ZIKV can be detected in amniotic fluid of fetuses with microcephaly substantiated this point.^19^

With the establishment of a prevention and control system for emerging infectious diseases in China after the outbreak of Severe Acute Respiratory Syndrome in 2003, imported cases from affected areas with fever will be strictly examined for specific pathogen infections. Travelers with fever would usually be intercepted for further quarantine in an international airport when they have a history of being bitten by a mosquito in an area affected by ZIKV. In our study, when the youngest son had a fever, the whole family needed to be examined. Because the four subjects in this family showed ZIKV positivity in their peripheral blood, isolation of the whole family was obligatory.

South American countries closest to the equator have been deeply plagued by ZIKV infection in recent years. For example, ZIKV infection has been frequently reported in counties such as Brazil, Suriname, Colombia and Venezuela.^10^ Neighboring Caribbean countries including Guatemala, Haiti, Puerto Rico and Dominica have also been affected. Throughout the whole year, these areas have a warm and humid climate suitable for the survival of mosquitoes. The temperature in February in North America, especially northern regions such as New York, is below 10 °C; thus, the family members in our study were unlikely to be bitten by mosquitoes during their 4-day stay in New York. Thus, the ZIKV isolates carried by this family could only have come from Venezuela (Figure 1).

Rash and low-grade fever seem to be the main symptoms for most ZIKV-infected individuals. Rash was reported to be the most frequent symptom (presented in 95.7% cases), followed by fever and arthralgia.^20^ In general, symptoms of ZIKV infection (mild flu-like symptoms) are milder than those of Dengue virus (DENV) infection; the latter often include high-grade fever with myalgia, headache, arthralgia and nausea. Leukopenia (<4000/mm^3^) has been detected in ~30% of Dengue fever patients.^21^ In our study, one adult and two children had the symptoms of fever, rash and conjunctival congestion, and no one complained of any pain. Unexpectedly, the three patients with fever all had normal leukocyte counts (>4000/mm^3^). Whether the milder symptoms of ZIKV infection (milder than DENV infection) mean a higher leukocyte count is unclear because of the lack of data obtained in past reports on ZIKV outbreaks. Hence, if the mother without any symptoms in our study is considered only a carrier of ZIKV (not a patient), this proportion of patients in our study is almost consistent with previous reports, in which rashes were presented by almost all patients.^20^ Information about pruritus, the second most common clinical symptom in the confirmed cases in previous study,^22^ was not obtained from the family while inquiring them regarding their case history. Perhaps this symptom is transient and unnoticeable at the time of disease onset.

RT-PCR based on TaqMAN probes may be the most convenient tool to detect ZIKV infection in suspected patients^23, 24^ because enzyme-linked immunosorbent assays (ELISAs) for IgM antibody against ZIKV would cross-react with other flaviviruses,^25, 26^ and the results must be validated by a plaque reduction neutralization test, a labor-intensive and costly method.^27^ Moreover, compared with blood drawing, urine and saliva collection are more acceptable options for suspected individuals, and the latter have an advantage for viral detection and isolation because virus can be detected at higher titers and for a longer period in urine than in serum.^28^ A previous study showed that a patient had prolonged shedding of viral RNA in saliva and urine for up to 29 days after symptom onset.^29^ Nevertheless, positive TaqMAN RT-PCR detection is not usually a guarantee of successful sequencing or isolation of target viruses. Urine and saliva usually need to be concentrated before RNA extraction or viral inoculation, which was performed in our study.

Like other flaviviruses, the structure of the single polyprotein encoded by ZIKV genomic RNA is 5′-C-prM-E-NS1-NS2A-NS2BNS3-NS4A-NS4B-NS5-3′, in which the E (envelope) protein is the main antigen recognized by the host immune system, and NS1 is the principal component of the replication complex of the virus. In previous research, E gene sequences of ZIKV isolates were usually utilized to construct phylogenetic trees^19^ based on experience from molecular study of dengue virus.^30^

Our phylogenetic tree constructed based on the E protein coding gene is topologically similar to that based on the complete ZIKV genomic sequence (Figure 4),^31^ meaning that the phylogenetic tree based on the E gene is strong enough to distinguish all clusters of ZIKV isolates from all over the world. Because complete genome sequencing of an RNA virus is a time-consuming procedure, constructing a phylogenetic tree using E protein gene sequences is a simpler method for molecular epidemiologic study, especially in an outbreak. In our phylogenetic tree constructed from E protein gene sequences, ZIKV can be divided into two lineages, African and Asian.^32, 33^ Although there is a very short evolutionary distance between our strains and the Venezuela imported strain KU744693.1-VE Ganxian, the diversity of the strains is apparent, and the latter is nearer to KJ776791.1-H/PF/2013 French Polynesian strains.^31, 34^ Currently, all cases reported in China seem to be imported from South America and Pacific islands, which also indicates there was a great diversity of strains circulating during early 2016 in Venezuela. Our strains are more similar to the strains circulating in South America in late 2015, where there were more strains isolated from patients. A phylogenetic tree based on NS1 gene sequences cannot efficiently distinguish different subgroups of ZIKV from various sources of isolates (Supplementary Figure S1) because the NS1 gene sequence is more conserved than that of E or even NS5, which is a gene sequence used more frequently to build phylogenetic trees. Currently, the E gene sequence is the most commonly used sequence in molecular epidemiologic studies of flaviviruses because it has the greatest genetic diversity (Supplementary Figure S1).

It is worth noting that strains of the African lineage were almost solely isolated from *Aedes* mosquitoes circulating in the African continent according to the phylogenetic tree, probably because ZIKV did not draw much attention before massive microcephaly cases were reported in Brazil. ZIKV is spread primarily by *A. aegypti*, a dominant vector for several mosquito-borne viruses in the Western Hemisphere.^35^ ZIKV was detected in *A. albopictus* from Gabon, a country in Central Africa.^9^ No direct evidence has demonstrated that this virus could be spread by *A. albopictus*, a local vector for Dengue virus in South China, yet the threat is quite real. The fact that ZIKV can propagate in the C6/36 cell line derived from *A. albopictus* suggests that this mosquito may be a competent vector and have the potential for spreading the virus,^5^ although there have not been any reported isolates from mosquitoes in South China. A previous experiment conducted in laboratory indicated that ZIKV could be detected in the midgut and salivary glands of *A. albopictus* ten days after oral infection.^36^ Outbreaks of DENV have proved that South China may be a permissible environment for transmission of emerging viral diseases such as ZIKV, and imported cases may be sources of subsequent autochthonous cases.^37, 38^

A key difficulty in preventing the spread of infection is that a high proportion of infected individuals have no symptoms. Similar to the mother in our study, asymptomatic individuals with viremia may be a source of infection when they are exposed to competent vectors. If the son's fever had not been found at the airport entrance, our subjects would not have been suspected of being infected with ZIKV, and thus their viremia would not have been confirmed. It is difficult to convince ZIKV carriers to be examined even if they are returning from an affected region. Furthermore, in addition to the mosquito-borne route, other complex modes of transmission such as body fluid transmission will make controlling ZIKV infection even more difficult than controlling Dengue fever. The priorities for control include strengthening the education of travelers from infected areas to non-epidemic regions, identifying virus carriers, preventing local transmission caused by imported cases, and an effective vector management program.

In our study, four subjects in one family had lived and traveled together for the past few months, so they had similar opportunities for exposure to mosquito bites. However, because they are a family, the probability of human-to-human transmission by close contact between family members should not be ignored. If their ZIKV infections were all caused by mosquito-biting, from a time axis showing when symptoms occurred, the incubation periods of three of the patients were all more than five days (Figure 3), which is consistent with previous reports (2–7-day incubation period according to the World Health Organization website). DENV transmission by non-blood routes is almost impossible, so vector transmission is nearly the only way. Thus, as for clustered cases in one family, if the vector transmission is considered, ZIKV can be comparable to DENV because they have the same transmission dynamics via vectors. Until now, there have been no reports of DENV case numbers in one family being more than two, especially when they share the same virus sequence. Therefore, because vector transmission of DENV between family members is less likely, so is that of ZIKV. Because the ZIKV isolated from three members is identical and there was a 1 to 3 day interval between initial symptom occurrence, human-to-human transmission may also be a plausible explanation. Moreover, viral isolation from urine and saliva samples (collected on 29 February 2016) of three patients was performed successfully using inoculation of suckling mice (data not shown), indicating that during onset, there was viral shedding in these three patients' body fluids, which was infectious to their family members.

Regarding direct transmission, sexual contact may be a route, for a relative case had been reported previously but with no direct evidence.^12^ Because an *in vitro* experiment demonstrated that human skin cells are permissive to ZIKV,^6^ it might be possible that ZIKV can be transmitted by other bodily fluids such as saliva and urine. RT-PCR positivity for ZIKV RNA together with successful viral isolation from the three patients' urine samples proved that there were live viruses present in these samples. RT-PCR also indicated the existence of viral RNA in the patients' saliva. However, currently there is no reliable evidence demonstrating ZIKV transmission via these types of bodily fluids.

## Figures and Tables

**Table 1:** The clinical features and laboratory tests from the patients with ZIKV infection

	**Infected individuals**			
**Characteristic**	**Father (patient 1)**	**Mother (patient 2)**	**Daughter (patient 3)**	**Son (patient 4)**
Ages (years)	40	37	8	6
Ethnic origin	Chinese	Chinese	Chinese	Chinese
History of living in infected area	+	+	+	+
History of mosquito bites	+	Not clear	+	+
Days of hospitalization	14	14	14	14
Clinical features				
Fever	−	−	+	+
Nausea	−	−	−	−
Vomiting	−	−	−	−
Diarrhea	−	−	−	−
Rash	+	−	+	+
Pharyngeal congestion	+	−	+	+
Conjunctival congestion	+	−		
Aches	−	−	−	−
Neurologic disorders	−	−	−	−
Laboratory tests^a^				
Blood routine analysis	Normal (1/3)	NA	Normal (26/2, 27/2)	Normal (26/2, 28/2,1/3)
Biochemical test	Normal (1/3)	NA	Normal (26/2, 27/2)	Normal (26/2, 28/2,1/3)
Routine urinalysis	NA	NA	NA	Normal (26/2)
ZIKV nuclear acid in blood (RT-PCR)	+	+	+	+
ZIKV nuclear acid in urine (RT-PCR)	+	+	+	+
ZIKV nuclear acid in saliva (RT-PCR)	+	+	+	+

Abbreviations: +, present; −, absent; not available, NA; Zika virus, ZIKV; real-time reverse transcription-polymerase chain reaction, RT-PCR.

a Some patients received multiple test, and date is indicated in brackets.
