# Supplementary material for: Epidemiologic investigation of a family cluster of imported ZIKV cases in Guangdong, China: probable human-to-human transmission
Source: Emerg Microbes Infect. 2016 Sep 7;5(9):e100–. doi: 10.1038/emi.2016.100 (PMC5113051; doi:10.1038/emi.2016.100)
Supplement: Supplementary Figure S1 [file emi2016100x1.doc]

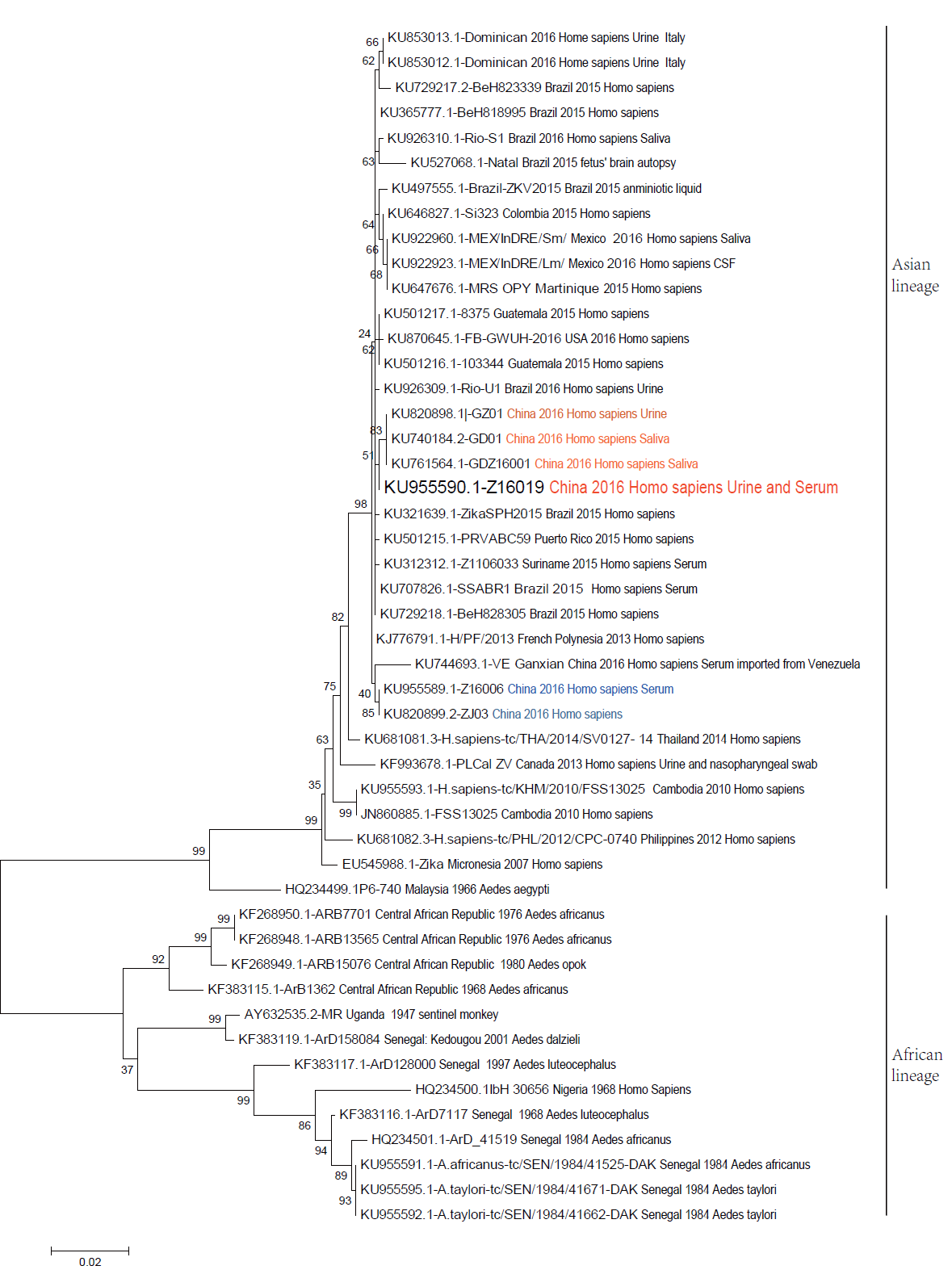


**Supplementary Figure S1** Phylogenetic tree based on nonstructural protein 1 (NS1) gene sequences of Zika virus isolates. NS1 gene sequence of the father (Genbank: KU955590) in our study was used to align with other reference sequences. All isolates is indicated with Genbank number, country, year and host (Genbank number, country, year and host, some with sample type). Phylogenetic trees were drawn using the Maximum likelihood method by Tamura-Nei model with gamma-distributed evolutionary rates in MEGA 7.0. Initial tree was made automatically with Nearest-Neighbor-Interchange (NNI) method. Gaps/missing data treatment was set as complete deletion. Bootstrap analyses with 1,000 replications were utilized to determine confidence values for groupings within the phylogenetic trees. Other parameters were set by a default style.
