# Supplementary material for: Epidemiologic investigation of a family cluster of imported ZIKV cases in Guangdong, China: probable human-to-human transmission
Source: Emerg Microbes Infect. 2016 Sep 7;5(9):e100–. doi: 10.1038/emi.2016.100 (PMC5113051; doi:10.1038/emi.2016.100)
Supplement: Supplementary Table 1 [file emi2016100x2.doc]

**Supplementary Table S1. Primer pairs for sequencing the region covering E and NS1 gene**

| Primer names | Primer sequences | Length of PCR products (bp) |
| --- | --- | --- |
| P1F1 | 5’-GCTCCCTTCCCATTCCACT-3’ | 960 |
| P1R1 | 5’-GCGTCCTTGAACTCTACCAG-3’ |
| P2F1 | CCAATTCACCAAGAGCCGAA | 964 |
| P2R1 | GGATAAGAAGATCAACACTCCCC |
| P3F1 | CGCTCTCAACTCATTGGGCAAG | 1019 |
| P3R1 | CCTCAAACCGAATTTCAAGCTC |
| P4F1 | GCCCACTCAAACATAGAGCAT | 1046 |
| P4R1 | GTGGAACAACCATCGCTCGT |
